# Weight Bias in Nursing: A Pilot Study on Feasibility and Negative Attitude Assessment Among Primary Care Nurses

**DOI:** 10.3390/nursrep15050168

**Published:** 2025-05-12

**Authors:** Jordi Benítez-Muñoz, María Jesús Aguarón-García, Maria del Carmen Malagón-Aguilera, Roser Cuesta-Martínez, Gloria Reig-Garcia, Maria Elena Solà-Miravete

**Affiliations:** 1 Department of Nursing, Rovira i Virgili University, 43002 Tarragona, Spain; mariajesus.aguaron@urv.cat (M.J.A.-G.); mariaroser.cuesta@urv.cat (R.C.-M.); mariaelena.sola@urv.cat (M.E.S.-M.); 2 Health and Health Care Research Group, Faculty of Nursing, University of Girona, 17004 Girona, Spain; carme.malagon@udg.edu (M.d.C.M.-A.); gloria.reig@udg.edu (G.R.-G.)

**Keywords:** nurse–patient relations, obesity, weight prejudice, perceived discrimination, empathy, nursing

## Abstract

**Background:** Weight bias in healthcare can affect the quality of care and create health disparities. In nursing, the presence of weight-biassed attitudes influences the therapeutic relationship and clinical decision-making. However, in Spain, research on this phenomenon remains scarce, hindering the development of strategies to mitigate its impact. **Objectives:** This study aimed to assess the methodological feasibility of a study on weight bias in nursing, and to explore nurses’ attitudes towards being overweight and obesity and their association with sociodemographic and body image variables. **Methods:** A cross-sectional, quantitative pilot study was conducted with 22 primary care nurses. The Anti-Fat Attitudes (AFA) and Beliefs About Obese Persons (BAOP) scales, previously validated in Spanish-speaking populations, were applied. Response distribution, the internal consistency of the instruments, and the relationship between variables were analysed. **Results:** Difficulties were identified in the recruitment of participants and the reliability of certain items of the questionnaire, as well as in the internal consistency of the scales. A trend towards moderate weight-biassed attitudes was observed in the sample, with the highest scores in the AFA’s “*Willpower*” subscale. The BAOP scale showed a significant negative correlation with the AFA (*r* = −0.55, *p* = 0.009), indicating that a lower attribution of obesity to individual control is associated with less discriminatory attitudes. **Conclusions:** This pilot study helped identify methodological improvements and confirmed the presence of weight bias in nursing. It is recommended that the sample be expanded and the measuring instruments refined before performing the full study.

## 1. Introduction

Weight bias in healthcare is a phenomenon that has been increasingly studied in recent years due to its impact on the quality of care and the health of people who are overweight or obese [1]. It is defined as a set of discriminatory attitudes, beliefs and practices within healthcare settings that perpetuate weight stigma, which can negatively affect the therapeutic relationship and care received by patients [2]. Although obesity has been identified as a risk factor for various pathologies, it is not always synonymous with disease, and taking a weight-centred approach has been criticised for reducing health to a single biometric indicator [3].

Different studies have documented how medical fatphobia can generate barriers to accessing healthcare, delays in receiving diagnoses, inappropriate treatments and negative experiences for patients [1,4,5]. People who are overweight and obese frequently report having been subjected to biassed clinical judgments, receiving weight loss advice as a general solution to any health problem regardless of the actual cause of their discomfort, which can even lead to the avoidance of health services, distrust in professionals and poor adherence [6,7]. Furthermore, research related to the topic has detected strong implicit and explicit fatphobic attitudes among doctors, nurses, nutritionists and dietitians [8,9,10]. With regard to primary and community care, studies primarily focus on medical staff but also find high rates of negative attitudes and stereotypes among this group towards people who are overweight or obese [11,12,13]. This discrimination not only has physical consequences but also psychological ones, affecting self-esteem, emotional well-being and trust in healthcare professionals.

In nursing, where interaction with patients is key to both health promotion and adherence to treatments, weight-based prejudice can influence clinical decision-making and the quality of care provided. Implicit bias can lead nurses to underestimate or overestimate the severity of certain conditions based on a patient’s weight, so affecting equity in care [14,15,16]. In this respect, Goad et al. suggest that variables such as Body Mass Index (BMI) may be related to the presence of negative attitudes towards people who are obese [17]. Moreover, the literature suggests that there is a need for nurses to improve their skills in areas such as empathy and communication in order to eliminate weight bias and discriminatory behaviours towards people who are overweight or obese [18].

Despite its importance, research on weight bias in nursing remains limited, especially in Spain [19,20], underscoring the need to address this issue from an empirical and contextualised perspective. In Spain, more than 60% of the population suffers from obesity or is at risk of suffering from it [21]. The SEEDO network reports that 66% of people with obesity have experienced weight stigma from society at large (driven by the perception of obesity as a lack of will and negative stereotypes) and this manifests itself in everyday situations such as shopping, in restaurants, or in the workplace, where people feel judged or observed. However, a significant source of stigma is healthcare professionals, with 58% of participants reporting negative experiences in this environment. This is linked to prejudices and negative attitudes on the part of professionals, including the belief that obesity is solely an individual responsibility, which affects the quality of care, diagnosis and treatment [21]. Although studies and research on medical fatphobia have intensified in recent years, they have a far greater presence in the international arena than they do in our home country, where we find few current examples beyond research carried out among nursing students [19,22].

This study was conceived as a pilot study to assess the methodological feasibility of a broader future study on medical fatphobia among primary and community care nurses. The primary objective is twofold. Firstly, it aims to analyse the feasibility of the study design, the appropriateness of the measurement instruments and data collection, allowing for the identification of possible adjustments that should be made before its larger-scale implementation. Secondly, it aims to find out whether nurses have negative attitudes towards overweight and obese patients and the relationship that these attitudes have with sociodemographic variables and the weight and body image of nurses.

## 2. Materials and Methods

### 2.1. Design

This study used a cross-sectional descriptive observational design carried out during the first quarter of 2024.

### 2.2. Sample

All 31 primary healthcare nurses employed at the Primary Care Centre in the Girona Health Region (Spain) were invited to participate using a non-probabilistic snowball sampling method. The inclusion criteria required nurses to be actively practicing during the study period and to voluntarily agree to participate. No additional inclusion criteria (for example, minimum years of experience) were applied, given the exploratory nature and small, homogeneous population of this pilot study.

Using the Granmo calculator (v. 8.0) for our finite population of 31 nurses—with a 95% confidence level and maximum variance assumption (*p* = 0.5)—we determined that 29 completed questionnaires were needed.

### 2.3. Instruments

For data collection, an ad hoc self-report questionnaire was designed, which nurses completed anonymously online using the Microsoft Forms platform. The link was sent via an internal distribution list that included all eligible nurses at the Centre. This questionnaire was accompanied by information regarding the study project, information on its proper conduct and information regarding confidentiality, anonymity and informed consent.

The questionnaire consisted of questions related to sociodemographic variables, such as age, gender, civil status, number of children, educational level, place and field of work and years of experience, as well as anthropometric variables, such as weight, height and body image perception according to Stunkard’s silhouettes scale [23]. Questions related to negative attitudes and behaviour towards overweight and obese people were formulated using the Anti-Fat Attitudes Scale, which is a validated version of the Anti-Fat Attitudes Scale (AFA) that is adapted to Spanish [24], with the approval of the authors for its use. This scale contains 13 items in 3 subscales. The first subscale is the “Dislike” subscale, which consists of 7 items and assesses whether respondents have negative feelings towards people who are overweight (e.g., “I find it hard to take a fat person seriously”). The second is the “fear of obesity” subscale, which consists of 3 items that assess the fear of gaining weight (e.g., “I feel annoyed with myself when I gain a little weight”). Finally, the “Willpower” subscale includes 3 items related to the perception that obesity is controllable (e.g., “some people are overweight because they lack willpower”). A Likert-type scale was used, with numerical responses ranging from 1 (“I do not agree at all”) to 7 (“I completely agree”). The final score is obtained by calculating the average of all responses for each subscale as well as the total score overall. High scores on this scale are associated with strongly negative attitudes towards people being overweight and obese. The form also included questions related to beliefs about the causes of obesity, taken from the Beliefs About Obese Persons Scale, which is a validated version of the Beliefs About Obese Persons Scale (BAOP) adapted to Spanish [25], with the approval of the authors for its use. This scale contains eight items with specific statements about the causes of obesity (e.g., “obese people eat more than non-obese people”). Respondents are asked to indicate the degree to which they identify with each statement using a 6-point Likert-type scale ranging from −3 (“I strongly disagree”) to +3 (“I strongly agree”). A high score indicates a stronger belief that obese people have little control over their body weight.

This pilot study was conducted in order to evaluate adaptation and applicability, with the aim of detecting possible difficulties that the nurses might have in understanding the items or in the data-gathering process. Furthermore, internal reliability was analysed by calculating Cronbach’s alpha to ensure the consistency of the scales in the study population. A preliminary data analysis was also carried out to identify potential response biases, and the logistical feasibility of the process was assessed taking into consideration aspects such as response time, participation rate and data collection efficiency.

The variables that were used for methodological feasibility were the response rate, the average time spent in responding to the instruments and cases lost due to non-response to different instrument variables.

### 2.4. Data Analyses

The statistical analysis was performed with Jamovi (version 2.6.25). Central tendencies and dispersion were calculated for quantitative variables, and frequencies and percentages for qualitative variables. Student’s t-test and the Mann–Whitney U test were used to compare groups depending on whether they followed a normal distribution, and ANOVA was used to analyse differences between three or more categories. The relationship between variables was assessed using the Pearson correlation coefficient.

### 2.5. Ethical Considerations

This study was approved by the People, Society and Environment Research Ethics Committee of the University of Rovira i Virgili of Tarragona (CEIPSA-2024-TFM-0098). The researchers informed all the participants about the objective of the study. The information collected through the questionnaires did not contain any personal data that could identify these participants, thus maintaining anonymity at all times. Participants were informed that their participation did not entail any risk. The data were analysed by a researcher and the principles defined in the Declaration of Helsinki were followed. The platform used to gather the study data complies with the security and data protection requirements established by current regulations, including the General Data Protection Regulation (GDPR) of the European Union and applicable national legislation. All responses were anonymised and could only be accessed by authorised researchers, guaranteeing the confidentiality and privacy of the participants.

## 3. Results

A total of 22 nurses from primary care and community health settings completed the questionnaire, representing a response rate of 71%.

The mean age of the nurses was 38.8 years (SD = 10.9), with a range of 23 to 56 years. Most identified as women (90.9%) and 9.1% identified as men (a non-binary option was offered but not selected by any participant). A total of 72.7% had completed postgraduate, master’s, or doctoral nursing studies; 81.8% worked in adult care, whereas 18.2% worked in paediatrics; and 81.8% had more than 10 years of work experience (Table 1).

With regard to weight and body perception, 81% of the nurses had a Body Mass Index (BMI) within the normal weight category, whereas 19% were overweight. None of the nurses had a BMI falling in the category of obese. The mean BMI was 23.1 (SD: 2.39). A total of 61.9% showed a distorted perception of their body image, primarily overestimating their BMI. Furthermore, 45.5% reported having experienced some form of fatphobic behaviour in their lives, while 9.1% had experienced it on several occasions (Table 2).

Table 3 shows the total scores obtained from the AFA and BAOP scales administered to the nurses. The AFA scale had a mean total score of 30.68 (SD: 10.06), suggesting negative attitudes towards obesity, with a maximum score of 56 and a minimum of 17. With regard to the AFA subscale scores, the Dislike subscale had a median score of 11.5 (IQR: 5); the Fear of Fat subscale had a mean score of 10.2 (SD: 5.18); and the Willpower subscale had a mean score of 9.18 (SD: 4.57). In the case of the BAOP scale, the mean total score was 26.7 (SD: 10.3), with a maximum score of 44 and a minimum of 8.

The differences in scores by variables are shown in Table 3. By gender, male nurses had a higher total score on the AFA scale (median: 47.5; IQR: 8.5), whereas the total score for female nurses was lower on the AFA scale (median: 29.5; IQR: 17) and higher on the BAOP scale (mean: 27.40; SD: 10.52). With regard to BMI, nurses with normal weight scored higher on the AFA scale (mean: 31.65; SD: 10.15) and lower on the BAOP scale (mean: 25.41; SD: 9.72), whereas overweight nurses scored lower on the AFA scale (mean: 24.75; SD: 9.46) and higher on the BAOP scale (mean: 35; SD: 9.8). In the case of having experienced weight discrimination, the total scores on the AFA scale were higher for nurses who reported having experienced discrimination several times (mean: 39.50; SD: 2.12) and higher on the BAOP scale (mean: 28; SD: 14.14) (Table 3).

With regard to the AFA subscale scores, gender differences were found in the Willpower subscale, where male nurses obtained higher scores than female nurses (median = 19.5; IQR = 1.50 and median = 9.0; IQR = 6, respectively), with statistically significant differences (*p* = 0.025). In the Fear of Fat subscale, nurses who had repeatedly experienced discrimination related to their weight obtained much higher scores (mean: 21.00; SD: 0), also with significant differences (*p* = 0.002) (Table 4). No statistically significant differences were found in the analysis of the remaining variables.

The correlation between the AFA and BAOP scales was negative according to the Pearson correlation coefficient (*r* = −0.55). This indicates that as the AFA scale score increases (reflecting greater fatphobia), the BAOP scale score decreases (indicating a greater belief that obesity is the exclusive result of individual factors such as lack of willpower); this correlation was statistically significant (*p* = 0.009). Likewise, a moderate negative correlation was found between the AFA Willpower subscale and the BAOP scale (*r* = −0.63), implying that those nurses who attribute obesity to a lack of self-control tend to reject multifactorial explanations for its origin (*p* = 0.002) (Table 5).

The results regarding methodological feasibility highlighted several issues that need to be taken into account. First, in the recruitment of nurses through the snowball technique, fewer nurses were obtained than required by the sample calculation. Furthermore, the limited presence of men in the sample constitutes a potential source of bias since male experiences and perceptions may be underrepresented in the results. The average time to complete the online questionnaire was 13 min and 18 s and no difficulties in understanding the items were reported during data collection. This information was gathered informally through oral feedback during the recruitment phase, during which participants were invited to share any issues or concerns regarding the content or clarity of the items. With regard to the loss of cases due to a failure to provide variables, the results were positive; only in one case were the weight and height variables not provided (*n* = 1 of 22; 4.5% missing data).

As far as the internal consistency of the instruments is concerned, the Spanish version of the AFA scale has an internal consistency of 0.85 as measured by Cronbach’s alpha coefficient [24]. The Cronbach’s alpha for the AFA scale for the sample studied was 0.56. As for the Spanish version of the BAOP scale, it has a Cronbach’s alpha coefficient of 0.60 for the whole scale [25]. For our sample, a Cronbach’s alpha coefficient of 0.74 was obtained for this scale.

## 4. Discussion

Although the BAOP scale obtained a Cronbach’s alpha coefficient that suggests acceptable reliability, this was not the case for the AFA scale, which was weaker in this sample. This is likely attributable to the small, non-random sample size, which can lead to underestimation of internal consistency estimates. Taking this factor, additional tests might be considered to determine whether the elimination of any particular item might increase the scale’s reliability, as other researchers have explored in similar studies [22]. Furthermore, the average response time for the online questionnaire was longer than initially expected, so the suitability and appropriateness of some of the questions should be reviewed, even though no difficulties in understanding were reported. With regard to missing data in the case of some variables, this could be related to participant biases due to the sensitivity of certain topics and a desire to conform to social norms.

The results of the study reflect scores on the AFA scale that support previous findings on the presence of fatphobic attitudes among healthcare professionals, including nurses [26,27]. Although the AFA scale does not have a scaled score, it is interpreted that the higher the score, the greater the presence of discriminatory attitudes towards people who are overweight and obese [24].

Although studies in nursing that assess negative attitudes towards weight using the AFA and BAOP scales are scarce, the literature shows that healthcare professionals, in general, have discriminatory attitudes towards people who are overweight or obese [11,12,13]. Recent studies among medical students also report similar weight-biassed attitudes, highlighting the persistence of this stigma during healthcare training [28,29], and although international evidence is abundant, the literature focused on the Spanish context remains limited, especially in the field of nursing [19,22]. These behaviours are not far removed from those detected in other areas of our society, demonstrating that, as Navajas-Pertegás suggests, the existence of discrimination and prejudice towards obese people is structural [26]. Despite this diagnosis, training interventions to reduce weight bias in healthcare professionals remain scarce and often with a lack of consensus [30,31,32]. In fact, systematic reviews on the subject highlight the lack of solid research and clear approaches to effectively address the problem of bias and weight stigma in the health field, which contributes to the lack of a structured approach to the problem. Studies on interventions to reduce weight bias in nurses are also limited, with most studies focusing on nursing students rather than nurses [19]. There is also no clear focus on interventions, using most research a mixed methodology. Some of them place more emphasis on obesity management education to reduce weight bias, while others focus on increasing empathy and raising awareness through obesity simulation suits and experiences based on clinical simulation and first-person experiences [33,34,35,36,37]. This lack of structured interventions adapted to nursing practice reinforces the need to rigorously study and investigate this problem to design innovative, practical and sustainable training proposals that contribute to more ethical, empathetic and inclusive care.

Comparisons of results by AFA subscales (Dislike, Fear of Fat and Willpower) are also in line with the existing literature. As stated by Magallares and Morales, men tend to score higher on both the Dislike subscale [24] and the Willpower subscale. These results are in line with those obtained in this research, although this finding must be interpreted with extreme caution given the very small number of male nurses. Even so, for the Fear of Fat subscale, higher scores in women have been found, and this fear is often associated with the aesthetic canons established in our society and with aesthetic social pressure [27]. This is not the case, however, with the data obtained in the sample, where both nurses identifying as male and those identifying as female have practically equal scores on this subscale. Where there are significant differences for this scale is in relation to having suffered discrimination due to one’s own body image, where the higher scores can be understood as being a result of the nurses’ greater awareness of this issue.

In agreement with Piñeyro’s study, people with normal weight show greater discrimination [38]. This can be explained by their privileged social position, which distances them from the reality of discrimination faced by those with non-normative bodies. In line with this idea, the results show that nurses with normal weight obtained a total score on the AFA scale that was significantly higher than those who were overweight. With regard to the scores on the BAOP scale, it should be noted that the total score of the sample is significantly higher than another study conducted on the subject [25]. High scores on this scale indicate the belief that obesity is not under the control of the individual; correspondingly, lower scores are associated with discriminatory attitudes towards obesity. According to authors such as Cohen et al. and Nath, this fact is associated with personality traits such as lack of willpower, laziness, or other traits attributed to this group [39,40]. If we analyse the results by gender, we can observe that there is a notable difference in scores between the male and female genders.

The results are framed in a sociocultural context where thinness has become a normative ideal, promoted by both the media and the predominant biomedical discourse [41,42]. Furthermore, the scale scores reflect what Piñeyro called the Fatphobic Tripartite, an interconnection between aesthetics, morals and health that sustains fatphobia [38]. The persistent association between obesity and certain negative attributes, such as lack of self-control or willpower, laziness or carelessness reinforces a stigma that not only affects the social perception of weight but also influences the therapeutic relationship between nurses and patients. These assumptions are intrinsically related to stereotypes and are likely to aggravate stigmatising attitudes towards people with obesity, while ignoring other social determinants of health such as the social, economic, cultural or environmental factors that come into play in its genesis. In fact, it has been shown, as Bernier points out, that weight is far from being the result of strictly personal choices [43]. This structural discrimination is reflected in the tendency of nurses to score high on the “Willpower” subscale, which suggests that biases regarding individual weight control are deeply rooted. Furthermore, the negative correlation between the AFA and the BAOP confirms that professionals with a more deterministic view of obesity display more fatphobic attitudes.

Conducting this pilot study is a fundamental step to ensure that the main research can be carried out effectively, obtaining an initial overview of negative attitudes towards weight, minimising potential biases and optimising the methodology used. The findings show a tendency towards negative attitudes and prejudices towards people who are overweight or obese and are consistent with previous studies reporting negative attitudes and biases towards obese patients among medical students and other healthcare professionals [31,44,45,46,47,48]. These beliefs and attitudes can negatively affect healthcare, leading to errors in diagnosis, delays in treatment and negligent conduct and malpractice, and may even force members of this group to leave the healthcare system [44]. Furthermore, they suggest and underscore the importance of training in non-stigmatising approaches in nursing practice.

### 4.1. Implications for Practice

The presence of weight bias or negative attitudes and behaviours towards people who are overweight and obese is deeply rooted as a sociocultural construct within the collective imagination [26] and is also present within the healthcare and nursing community. Although being overweight and obesity, in general, have multiple causes, the individual is often held responsible for the situation, generating feelings of guilt that can often translate into emotional problems and psychological distress [49].

Assuming the full study confirms the findings of this initial pilot study regarding the existence of weight bias among primary care nurses, it will be possible to use the results to guide the implementation of training strategies to raise awareness and reduce this bias and these behaviours. Nursing teams can reduce weight bias and improve patient care through target, evidence-based training, as demonstrated in recent intervention studies [34,50,51,52,53]. Such an approach would help reduce the stigma surrounding obesity and its impact not only on the biological health of those affected but also on their emotional well-being.

It is believed that this is the first study to analyse the perceptions that nurses have of this issue. Knowledge of the initial situation will allow the effectiveness of future interventions in this area to be evaluated.

### 4.2. Limitations

The small sample size and lack of diversity in participant selection (only two male nurses) are important limitations of this study, as they may influence the generalisability of the results. The use of snowball sampling and the small, non-random sample could have led to an overrepresentation of individuals with certain attitudes, contributing to the observed gender imbalance that is observed. However, it is crucial to note that nursing is still a highly feminised profession, both globally and in the Spanish context. According to the Spanish National Institute of Statistics (INE), women make up the vast majority of registered nurses in Spain, with 84.1% of nurses being female in 2024 [54]. Therefore, the underrepresentation of male participants in our sample reflects the demographic profile of the profession, especially in small-scale studies. Although the low number of male participants limits the ability to analyse gender-based differences in depth, this is consistent with the population under study.

Furthermore, this study was designed as a preliminary, exploratory investigation to assess the methodological feasibility of examining weight bias in nursing within the Spanish context. The primary aim was not to produce generalisable findings but to identify initial trends, evaluate data collection procedures and highlight key issues for future, larger-scale studies. This approach is consistent with standard practices in pilot and feasibility research, where smaller, targeted samples are used to inform the design of more extensive investigations.

Moreover, the reliability of the AFA scale in this specific sample was lower than expected, which suggests the need for future adaptations or validations to increase its internal consistency if this is not related to the small sample size. A further concern is that some bias may occur in the responses related to the AFA and BAOP scales, as some participants may feel they are being accused or morally judged and may not answer the questions with complete frankness.

Despite these limitations, the pilot study has enabled the identification of areas for methodological improvement and partially validated the importance of the large-scale study. Future studies should focus on expanding the sample and designing specific awareness-raising interventions to reduce medical fatphobia in nursing.

## 5. Conclusions

With regard to methodological feasibility, this study identified difficulties in recruiting nurses to achieve the required sample size and in the relatively lengthy amount of time required to complete the questionnaire. Furthermore, the small and non-random nature of the sample limits the generalisability of these findings. Although missing data have been reported, the proportion is low and does not affect the validity of the findings. The reliability and internal consistency scores for some scale items were lower than expected due to the small sample size. The results presented here will allow the instruments to be adjusted and the methodology to be optimised before large-scale implementation is undertaken.

The results of the pilot study show the presence of moderate to high levels of negative attitudes towards obesity among the participating nurses. Furthermore, these negative attitudes were more pronounced among male nurses and among nurses classified as being of normal weight based on their BMI. Nurses who had experienced one or more episodes of discrimination based on their weight or body image were found to have greater sensitivity towards people being overweight or obese.

## Figures and Tables

**Table 1:** Sociodemographic characteristics of the sample.

	Total Study Population (*n:* 22)
Age (Mean, SD)	38.8 (10.9)
Gender	*n* (%)
Women	20 (90.9)
Men	2 (9.1)
Level of education related to the nursing profession	
RN	6 (27.3)
MSc	15 (68.2)
PhD	1 (4.5)
Employment field	
Adults	18 (81.8)
Paediatrics	4 (18.2)
Years of experience	
From 0 to 5	4 (18.2)
From 6 to 10	5 (22.7)
From 11 to 15	2 (9.1)
From 16 to 20	2 (9.1)
From 21 to 25	6 (27.3)
Over 25	3 (13.6)

Descriptive results: *n* (%). Frequency (percentage). RN: Registered Nurse.

**Table 2:** Nurses’ variables related to weight, image and discrimination.

	Total Study Population (*n:* 22)
Body Mass Index—BMI (Mean, SD)	23.9 (2.39)
BMI categories	*n* (%)
Normal weight	17 (81)
Overweight	4 (19)
Obesity	-
Lost data	1
BMI according to perception of body image (Median, IQR)	25 (3.50)
Difference between real BMI and perceived BMI (Mean, SD)	−2.34 (1.47) ^1^
Weight discrimination	
Never	10 (45.5)
Sometimes	10 (4.5.5)
Many times	2 (9.1)

Descriptive results: *n* (%). Frequency (percentage). ^1^: According to Stunkard’s silhouettes scale, negative scores below 2 indicate an overestimation of BMI.

**Table 3:** AFA and BAOP scale scores according to variables.

Scale Scores	Total Study Population (*n*: 22)		
	Mean; SD *	Median; IQR *	*p*
AFA Scale Total Score	30.68 (10.16)		
By gender			
Women		29.50 (17)	0.059
Men		47.5 (8.5)	
By BMI			
Normal weight	31.65 (10.15)		0.232
Overweight	24.75 (9.46)		
By discrimination			
Never	28 (9.14)		0.327
Sometimes	31.6 (11.23)		
Several times	39.5 (2.12)		
BAOP Scale total score	26.68 (10.33)		
By gender			
Women	27.40 (10.52)		0.314
Men	19.50 (4.95)		
By BMI			
Normal weight	25.41 (9.72)		0.080
Overweight	35 (9.8)		
By discrimination			
Never	24.7 (9.13)		0.732
Sometimes	28.4 (11.64)		
Several times	28 (14.14)		

* Mean or median was used depending on whether the data followed a normal distribution or not.

**Table 4:** AFA subscales scores according to variables.

Subscales of AFA (Range Score)	Total Study Population (*n*: 22)		
	Mean; SD *	Median; IQR *	*p*
AFA Dislike subscale (7–49)			
By gender			
Women		10.50 (5)	0.167
Men		17.5 (5)	
By BMI			
Normal weight		12 (4)	0.190
Overweight		8 (2.8)	
By discrimination			
Never	10.10 (3.48)		0.326
Sometimes	12.7 (1.22)		
Several times	10.50 (3.54)		
AFA Fear of fat subscale (3–21)			
By gender			
Women	10.15 (5.42)		0.930
Men	10.5 (2.12)		
By BMI			
Normal weight		9 (6)	0.368
Overweight		19.5 (1.5)	
By discrimination			
Never	8.3 (4.62)		0.002^†^
Sometimes	9.9 (3.35)		
Several times	21 (0)		
AFA Willpower subscale (3–21)			
By gender			
Women		9 (6)	0.025^†^
Men		19.5 (1.5)	
By BMI			
Normal weight	9.76 (4.71)		0.280
Overweight	6.5 (4.04)		
By discrimination			
Never	9.6 (3.95)		0.899
Sometimes	9 (5.4)		
Several times	8 (5.66)		

* Mean or median was used depending on whether the data followed a normal distribution or not.

**Table 5:** Pearson correlations between AFA-BAOP (also by subscales).

Scales		AFA	BAOP	*Dislike*	*Fear*	*Willpower*
AFA	*r*	-				
	*p*	-				
BAOP	*r*	−0.55 **				
	*p*	0.009				
*Dislike*	*r*	0.724 ***	−0.306			
	*p*	<0.001	0.166			
*Fear of Fat*	*r*	0.727 ***	−0.273	0.250		
	*p*	<0.001	0.219	0.261		
*Willpower*	*r*	0.763 ***	−0.633 **	0.460 *	0.254	-
	*p*	<0.001	0.002	0.031	0.254	-

Note: * *p* < 0.05, ** *p* < 0.01, *** *p* < 0.001.

## Data Availability

The data presented in this study are available on request from the corresponding author.

## Public Involvement Statement

There was no public involvement in any aspect of this research.

## Guidelines and Standards Statement

This manuscript was drafted against the Strengthening the Reporting of Observational Studies in Epidemiology (STROBE) Statement guidelines for reporting observational research.
